# A novel epithelial-mesenchymal transition (EMT)-related gene signature of predictive value for the survival outcomes in lung adenocarcinoma

**DOI:** 10.3389/fonc.2022.974614

**Published:** 2022-09-15

**Authors:** Yimeng Cui, Xin Wang, Lei Zhang, Wei Liu, Jinfeng Ning, Ruixue Gu, Yaowen Cui, Li Cai, Ying Xing

**Affiliations:** ^1^ The Fourth Department of Medical Oncology, Harbin Medical University Cancer Hospital, Harbin, China; ^2^ Department of Thoracic Surgery, Harbin Medical University Cancer Hospital, Harbin, China

**Keywords:** lung adenocarcinoma, EMT, signature, prognosis, nomogram

## Abstract

Lung adenocarcinoma (LUAD) is a remarkably heterogeneous and aggressive disease with dismal prognosis of patients. The identification of promising prognostic biomarkers might enable effective diagnosis and treatment of LUAD. Aberrant activation of epithelial-mesenchymal transition (EMT) is required for LUAD initiation, progression and metastasis. With the purpose of identifying a robust EMT-related gene signature (E-signature) to monitor the survival outcomes of LUAD patients. In The Cancer Genome Atlas (TCGA) database, least absolute shrinkage and selection operator (LASSO) analysis and cox regression analysis were conducted to acquire prognostic and EMT-related genes. A 4 EMT-related and prognostic gene signature, comprising dickkopf-like protein 1 (DKK1), lysyl oxidase-like 2 (LOXL2), matrix Gla protein (MGP) and slit guidance ligand 3 (SLIT3), was identified. By the usage of datum derived from TCGA database and Western blotting analysis, compared with adjacent tissue samples, DKK1 and LOXL2 protein expression in LUAD tissue samples were significantly higher, whereas the trend of MGP and SLIT3 expression were opposite. Concurrent with upregulation of epithelial markers and downregulation of mesenchymal markers, knockdown of DKK1 and LOXL2 impeded the migration and invasion of LUAD cells. Simultaneously, MGP and SLIT3 silencing promoted metastasis and induce EMT of LUAD cells. In the TCGA-LUAD set, receiver operating characteristic (ROC) analysis indicated that our risk model based on the identified E-signature was superior to those reported in literatures. Additionally, the E-signature carried robust prognostic significance. The validity of prediction in the E-signature was validated by the three independent datasets obtained from Gene Expression Omnibus (GEO) database. The probabilistic nomogram including the E-signature, pathological T stage and N stage was constructed and the nomogram demonstrated satisfactory discrimination and calibration. In LUAD patients, the E-signature risk score was associated with T stage, N stage, M stage and TNM stage. GSEA (gene set enrichment analysis) analysis indicated that the E-signature might be linked to the pathways including GLYCOLYSIS, MYC TARGETS, DNA REPAIR and so on. In conclusion, our study explored an innovative EMT based prognostic signature that might serve as a potential target for personalized and precision medicine.

## Introduction

Lung adenocarcinoma (LUAD), as the predominant histological type of lung cancer, has biological characteristics of strong aggressiveness and heterogeneity (1–3). In spite of optimized treatment methods including surgery, chemotherapy, radiotherapy, immunotherapy and targeted therapy, prognosis of LUAD patients remains dismal, because of cancer progression (4, 5). Collectively, it is imperative to distinguish populations at a high-risk of LUAD for early intervention and improving clinical outcome. At present, the combination of clinicopathological features and TNM staging system is the consensus criterion for determining treatment options and predicting relapse of LUAD, however this criterion restrains the provision of optimal clinical care to patients (6). Hence, identifying reliable biomarkers for optimizing the prognosis of LUAD is urgently needed.

Metastases are the primary cause of LUAD-associated mortality (7, 8). Migratory tumor cells escape from the primary site, remodel the basement membrane to engage with peritumoural stroma, undergo intravasation, endure shear stress in circulation and adapt to the tumor microenvironment of distant metastasis (9, 10). In total, the metastatic process generally involves three distinct phases: dissemination, dormancy and colonization (11). Epithelial mesenchymal transition (EMT) of cancer cells is a fundamental event during the multistep process participated in cancer metastasis (12, 13). Moreover, EMT activation confers on tumor cells to acquire plasticity with more aggressive phenotype, which exerts a decisive function on the malignant cancer progression (14, 15). Accumulating evidence has revealed that EMT process was closely implicated in tumorigenicity, angiogenesis and drug resistance (16–18). EMT results in a series of changes during epithelial tumor cells transforming into mesenchymal cells, including loss of tight junctions, cell polarity, cytoskeletal reorganization, and increase of cell viability (19). EMT is controlled or induced by a number of factors such as exosomal circRNAs, varied transcripts (Twist, Snail, ZEB1, et al.), microRNAs (miR212, mir200 family, et al.) and cellular oncogenic pathways (EGFR signaling pathway, et al.) (20–22). There is a significant link between the aberrant expression of genes related with EMT and poor clinical outcomes in LUAD patients (13, 23).

Since the advent of next generation sequencing, research on bioinformatic analysis has flourished (24; 25). For instance, The Cancer Genome Atlas (TCGA) database and Gene Expression Omnibus (GEO) database, such public databases are desirable to access transcriptomic information, that advances the efficient methods to select gene signatures (26–28). Numerous studies have attempted to construct the risk model to get biological characteristics or prognostic appraisal in malignant tumors, which had potential clinical impact (29–31). Interestingly, EMT-related gene signature (E-signature) could reveal the prognostic consequences in various types of cancer (32–34). Nevertheless, the existence of heterogeneity in samples among diversified studies on various tumours resulting in different risk models (35). Recently, some researchers reported the prognostic significance of E-signature, however they did not validate the biological functions of EMT-related genes with *in vitro* experiments in LUAD and their studies remains rudimentary to some extent (36–38).

In the current study, candidate genes involved in EMT process were identified based on TCGA-LUAD training dataset and validated using cell-based assays *in vitro* and clinical tissue samples. Then, the E-signature which can accurately predict the prognosis of LUAD patients was developed by us. Meanwhile, the nomogram affirmed the feasibility in clinical application of the E-signature. Subgroup analysis suggested that E-Signature could be conducive to identify patients with adverse events at high risk. The E-signature is closely related to multiple pathways associated with cancer progression, as well. In conclusion, the E-signature could be used as an inspiring molecular indicator for evaluation of clinical prognosis in LUAD patients.

## Materials and methods

### Data capturing and processing

**Figure 1:**
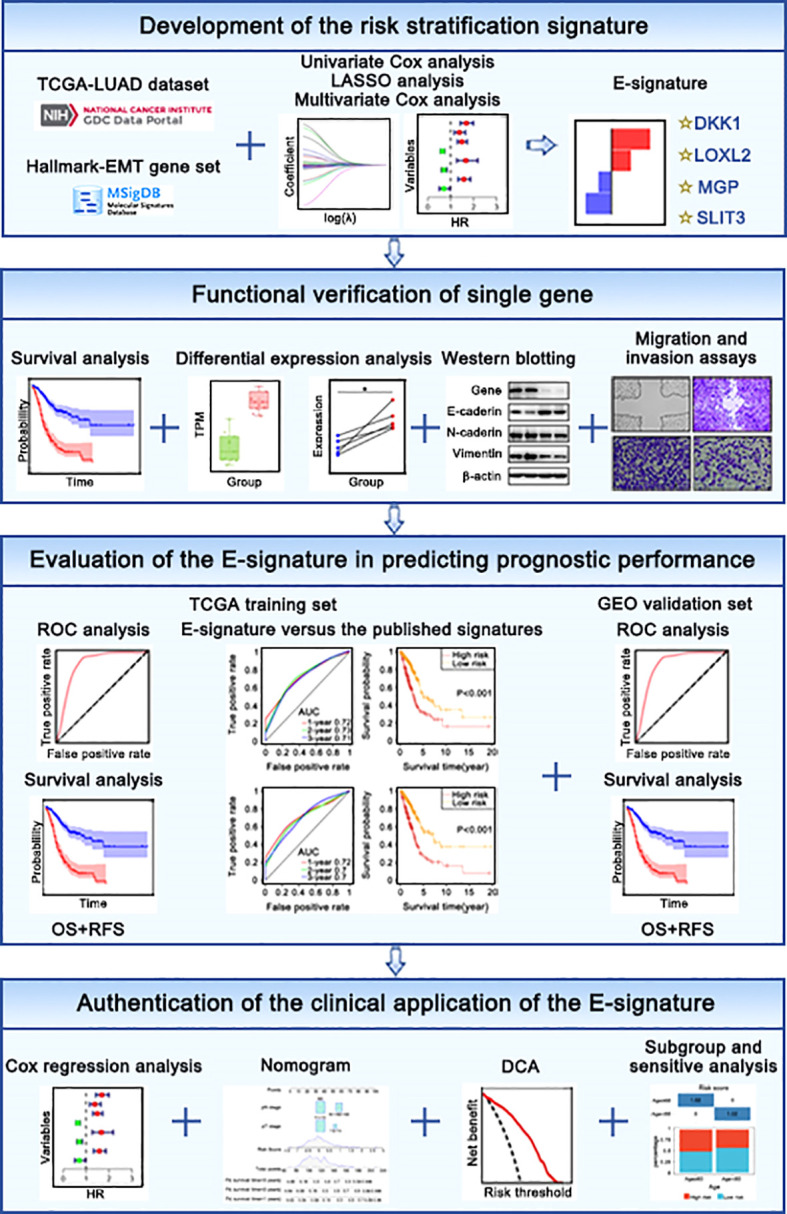
The flow chart in our study.

Entire design attached to our research was presented in **Figure 1**. TCGA website (http://portal.gdc.cancer.gov) were used to obtain raw microarray data and matched clinical of LUAD patients. Expression profiles were processed with robust multiarray average (RMA) algorithm (29). Differential expression analysis was performed by “limma” package. GEO database(https://www.ncbi.nlm.nih.gov/geo/) (**Table 1**) were used as independent external verification sets, including GSE30219, GSE37745, GSE50081 and GSE8894 (https://www.ncbi.nlm.nih.gov/geo/) (**Table 1**). Besides, we retrieved “HALLMARK EPITHELIAL MESENCHYMAL TRANSITION” gene list encompassing 200 genes in the MsigDB (https://www.gsea-msigdb.org/gsea/msigdb/index.jsp).

**Table 1:** Various clinicopathological characteristics of patients with LUAD in TCGA training cohort and GEO datasets.

Characteristics		TCGA Set (n = 490)	GSE30219 (n = 83)	GSE37745 (n = 105)	GSE50081 (n = 128)	GSE8894 (n = 63)
**Age(years)**	≤60	153	43	45	19	30
	>60	327	40	60	109	31
**Survival status**	Alive	312	40	29	76	–
	Dead	178	43	76	52	–
**Recurrence**	No	284	56	–	88	30
	Yes	206	27	–	37	29
**Gender**	Female	262	18	60	53	34
	Male	228	65	45	65	–
**pT stage**	T1	163	69	–	43	–
	T2	263	12	–	83	–
	T3	43	2	–	2	–
	T4	18	0	–	0	–
**pN stage**	N0	317	80	–	94	–
	N1	92	3	–	34	–
	N2	68	0	–	0	–
	N3/NX	12	0	–	0	–
**pM stage**	M0	324	83	–	128	–
	M1/MX	162	0	–	0	–
**Tumor stage**	Stage I	263	–	70	92	–
	Stage II	115	–	19	36	–
	Stage III	79	–	12	0	–
	Stage IV	25	–	4	0	–
**Smoking**	No	68	–	–	23	–
	Yes	408	–	–	92	–
**EGFR**	Wild type	186	–	–	–	–
	Mutant	79	–	–	–	–

### LASSO Cox regression analysis

To yield the independent prognostic EMT factor, univariate analysis and multivariate analysis were showed with *P<* 0.05 as a threshold. least absolute shrinkage and selection operator (LASSO) analysis was capable for reducing the dimension (39). 80% TCGA samples after preprocessing were randomized to the training dataset. our prognostic model preserved the advantage of subset shrinkage and maintained a high accuracy rate based on the penalty parameter *λ* (40). Risk score was calculated from expression of related gene and associated coefficient. Analytical formula for risk score assessment was derived on the basis of the EMT related gene signature = $\sum_{i=1}^{n}\text{(coef}i\;\times \text{Expr}i)$ , in this formula, Expr*_i_* represents gene expression of patient, and coef*_i_* represents the multivariate regression coefficient.

### Survival analysis

According to median risk scores, all LUAD samples were divided into two groups, involving in high-risk and low-risk groups in different cohorts. Kaplan Meier (KM) curves were plotted to compare the prognostic difference (41). The area under the curve (AUC) which performed from receiver operating characteristic (ROC) curve analysis for overall survival (OS) was employed to designate the predictive efficiency (42).

### Establishment and validation of the nomogram

The significant variables from the multivariate models were introduced to draw the graphical nomogram by utilizing “rms” and “nomogramEx” packages (43). The calibration curves for probability of OS showed that match condition between prediction by nomogram and actual observation (44). Decision curve analysis (DCA) was utilized to evaluate the ability of the predictive model in view of clinical applicability (44).

### Gene set variation analysis (GSVA)

Single-sample gene set enrichment analysis (ssGSEA) scores were gained by “ClusterProfiler” and “GSVA” R package to recognize the correlation between risk scores and enriched biological processes (45). The results with a cut-off criterion of *P* value< 0.05 were statistical significance.

### Collection of LUAD tissues and cell culture

Under the premise that ethical clearance and approval have been obtained, 5 pairs of fresh tumor samples and matched normal tumor-adjacent samples were dissected from 5 LUAD patients who underwent lobectomy but did not receive radiotherapy and chemotherapy at Harbin Medical University Cancer Hospital between May 2021 and June 2021.

Human LUAD cell lines (A549 and NCI-H1299) were cultured with RPMI-1640 including 10% fetal bovine serum (FBS) and 1% penicillin/streptomycin, and the culture environment needed to be maintained at 37°C and containing 5% carbon dioxide.

### Small interfering RNA transfection

SiRNAs of dickkopf-like protein 1 (DKK1), lysyl oxidase-like 2 (LOXL2), matrix Gla protein (MGP) and slit guidance ligand 3 (SLIT3) were made by Hanyinbt (Shanghai, China), in addition, these target sequences were presented as: DKK1#1, 5’-GCUUCACACUUGUCAGAGAtt-3’; DKK1#2, 5’-GGCUCUCAUGGACUAGAAAtt-3’; LOXL2#1, 5’-CAUACAAUACCAAAGUGUAtt-3’; LOXL2#2, 5’-GGGUGGAGGUGUACUAUGAtt-3’; MGP#1, 5’-CCCUACUGCUGCUACACAATT-3’; MGP#2, 5’-GAUAAGUAAUGAAAGUGCATT-3’; SLIT3#1, 5’-CGCGAUUUGGAGAUCCUUAtt-3’; SLIT3#2, 5’-GUACAAAGAGCCAGGAAUATT-3’. All corresponding negative control siRNA sequences were completed by Hanyinbt Company.

When the density of A549 and H1299 cells reached 60-70% in 6-well dishes, the transfection mixture was prepared by fully mixing 120μL riboFECT™CP Buffer, 12μL riboFECT™CP Reagent and 5μL siRNA, and incubated at room temperature for 15min. Finally, 3mL RPMI-1640 containing 10% FBS was added into the 6-well dishes. Follow-up experiments were carried out after ensuring the efficiency of knockout.

### Western blotting

Lysate of tissues or cells was centrifuged for 15 min (4°C, 14000rpm) and we measured protein concentrations using the BCA protein analysis kit. Electrophoretic separation and electro-transfer of protein samples with the comparable quality, membrane blocking and incubate overnight (primary antibody, 4°C). PVDF membranes containing proteins are incubated with secondary antibodies for 1 hour at room temperature. The bands on membrane were exposed by CL Xposure film (Thermo Fisher Scientific). Specific antibodies included: DKK1 (21112-1-AP, Proteintech Group Inc., Wuhan, China), LOXL2 (AB179810, Abcam), MGP (10734-1-AP, Proteintech Group Inc., Wuhan, China), SLIT3 (DF9909, Affinity Biosciences, Jiangsu, China), E-Cadherin (20874-1-AP, Proteintech Group Inc., Wuhan, China), N-Cadherin (22018-1-AP, Proteintech Group Inc., Wuhan, China), Vimentin (10366-1-AP, Proteintech Group Inc., Wuhan, China), β-actin (AF7018, Affinity Biosciences, Jiangsu, China).

### Detection of cancer cell migration and invasion

Use the tip of a 10 μL pipette to form a scratch on a six-well plate covered with LUAD cells. By comparing the rate of wound healing and taking pictures under the microscope, the cell migration rate was finally counted. Transwell assay was conducted with the Corning Inc. transwell chamber. The invasion experiment required the participation of the matrigel matrix involved. Cell suspension arranged in the upper chamber contained 2x10^4^ cells, besides, ingredient of the lower chamber was 600μL RPMI-1640 complemented with 10% FBS. The fixation of methanol and staining of 0.1% crystal violet for observing the migration and invasion efficiency after 24h or 48h, respectively. Fields were randomly selected in each membrane for capturing.

### Statistical methods

Data processing was focused on GraphPad Prism 8.0.2 software. Typicality of data in the study could be reflected with at least three independent experiments completed. Continuous data conforming to a normal distribution were analyzed by Student’s t-test, and subgroup differences were counted using the χ2 test. All results are expressed as mean ± SEM (* *P*< 0.05, ** *P*< 0.01, *** *P*< 0.001).

## Results

### The identification and validation with *in vitro* experiments of 4 prognostic EMT-related genes comprising the E-signature

**Figure 2:**
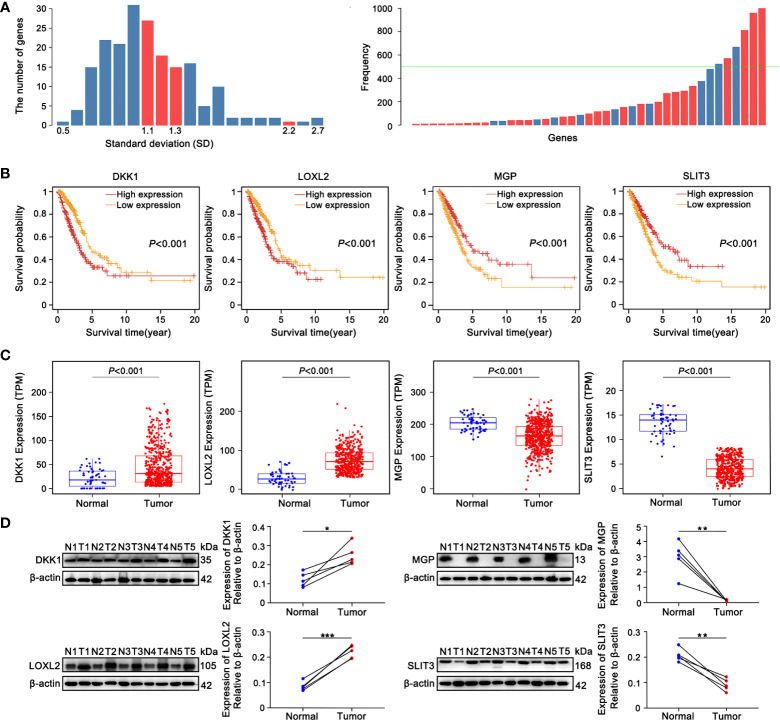
The identification 4 prognostic EMT-related genes comprising the E-signature. **(A)** The standard deviation distribution of all mRNAs. In the left panel, location corresponding to the red columns were standard deviation (SD) of genes with frequency greater than 500, where SD was used as the abscissa and the vertical axis represented the number of genes. The right panel showed that the gene frequency distribution chart obtained by LASSO analysis. The green baseline meant that frequency was equal to 500, the part exceeded the green line was genes with frequency greater than 500. **(B)** KM curves showed the overall survival (OS) of patients grouped according to expression patterns of 4 prognostic EMT-related genes comprising the E-signature in TCGA-LUAD datasets. **(C)** Differential expression analysis of DKK1, LOXL2, MGP and SLIT3 originated from TCGA-LUAD dataset. **(D)** The expression of 4 genes in fresh LUAD tumor samples (T) and adjacent normal-frozen tissues (N) detected by Western blot (*P< 0.05, **P< 0.01, ***P< 0.001).

The 200 genes linked to EMT were acquired from the “HALLMARK_EPITHELIAL_MESENCHYMAL_TRANSITION” gene list in the MsigDB. Univariate Cox analyses systematically deduced 68 genes significantly relevant to prognosis based on TCGA-LUAD training cohort. To exploit an optimal model for testing risk, the LASSO analysis was used in its broadest sense to summary the results of each dimensionality reduction and count the occurrence frequency and standard deviation distribution of each probe. Standard deviation (SD) calculations may comprehensively describe the distribution characteristics of data, which is generally understood to measure the deviation degree (46). Four EMT-related genes, including dickkopf-like protein 1 (DKK1), lysyl oxidase-like 2 (LOXL2), matrix Gla protein (MGP) and slit guidance ligand 3 (SLIT3), were comprehensively screened depending on the following criteria (**Figure 2A**). SD of candidate mRNAs was greater than the median and frequency was well over 500 (**Figure 2A**). Patients with high DKK1 and LOXL2 expression had the shorter overall survival (OS) according to a Kaplan-Meier analysis, whereas ones with high expression of MGP or SLIT3 had longer OS (**Figure 2B**). Based on TCGA-LUAD transcriptome data, differential expression analysis showed that with the comparison of normal samples, DKK1 and LOXL2 expression were significantly increased in tumor tissues, whereas MGP and SLIT3 were markedly overexpressed in non-tumoral tissues (**Figure 2C**). The same results were achieved from the collected clinical tissue samples by western blotting (**Figure 2D**).

**Figure 3:**
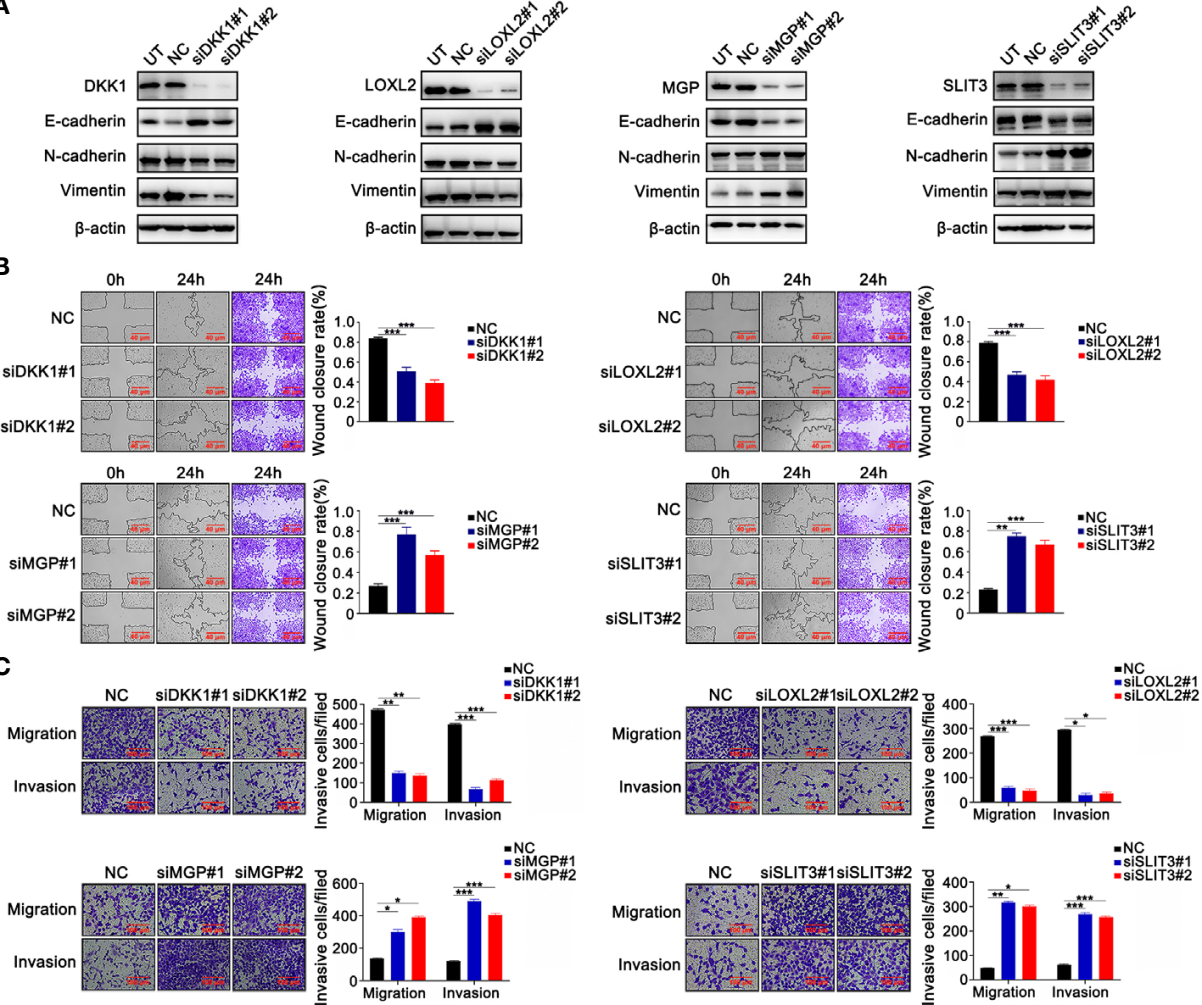
The validation experiments of 4 prognostic EMT-related genes comprising the E-signature *in vitro*. **(A)** Western blotting confirmed that silencing 4 genes respectively caused alterations of EMT‐related protein expression in H1299 cells. **(B)** The effect of silencing four genes on H1299 cell migration confirmed by wound-healing assays. **(C)** Transferability and invasiveness of H1299 cells were evaluated. *P < 0.05; **P < 0.01; ***P < 0.001. Data wereobtained from three independent experiments.

The functions and roles of these four genes in LUAD metastasis and EMT remain to be clarified (47–50). We used cell-based assays *in vitro* to examine the effect of these four genes on EMT and metastasis, respectively. In A549 and H1299 cells, Gene-specific siRNAs were used with three independent siRNAs for knockdown of each gene. Successful knockdown of these four genes was validated by Western blotting in A549 (**Figure 3A**) and NCI-H1299 cell lines (**Figure S1A**). Moreover, as illustrated in **Figure 3A** and **Figure S1A**, knockdown of DKK1 or si-LOXL2 enhanced the expression of E-cadherin and inhibited the mesenchymal markers’ expression, including N-cadherin and Vimentin. MGP or SLIT3 knockdown led to downregulation of epithelium-derived markers and upregulation of mesenchymal markers (**Figure 3A**; **Figure S1A**). Furthermore, wound healing and Transwell assays revealed silencing of DKK1 and LOXL2 suppressed the ability of migration and invasion in LUAD cells, but silencing of MGP and SLIT3 had the opposite effect (**Figures 3B, C**; **Figures S1B, C**). Our experimental data indicated that the four EMT-related genes could regulate metastasis and EMT program.

### Construction of the prognostic E-signature in LUAD

**Figure 4:**
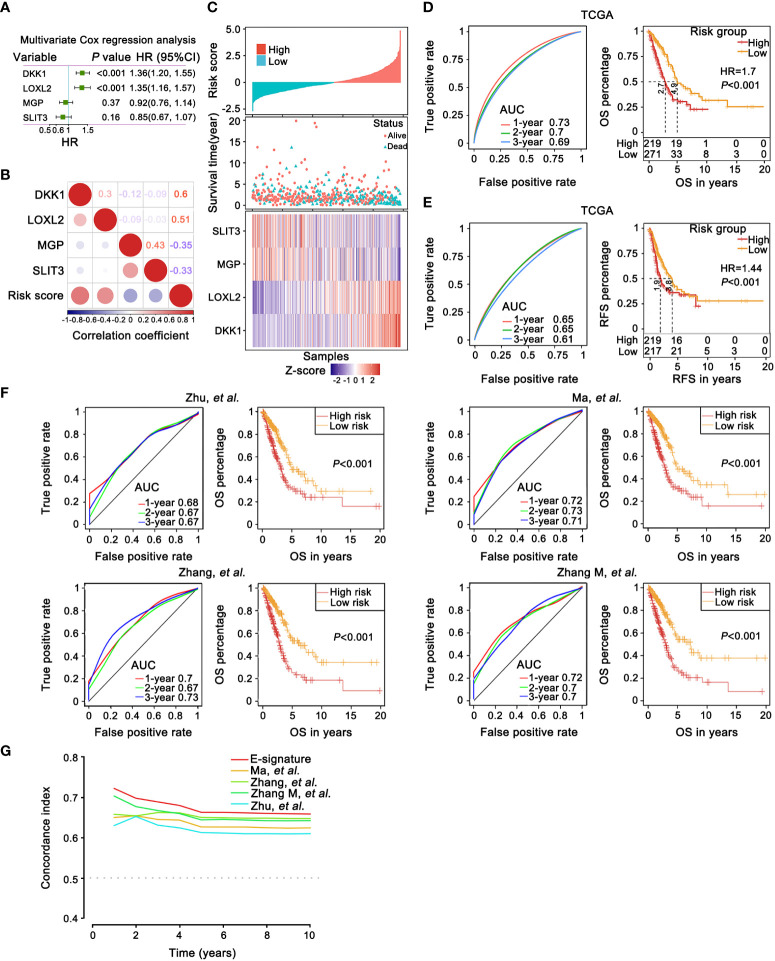
The prognostic robustness and clinical usefulness of the E-signature in the internal training set. **(A)** Evaluating the impact of 4 EMT-related genes on OS by means of forest plot. **(B)** Relatedness plot reported Pearson correlation values of each comparison. The bar color indicated Pearson corr. Values below the map calculated between risk score and genes in the matrix. **(C)** The risk score, survival time and status of each sample in TCGA-LUAD cohorts. The heatmap listed expression status of each gene involved in the signature. **(D, E)** The time-dependent receiver operating characteristic (ROC) curves for the 1-, 2- and 3-year OS **(D)** and relapse free survival (RFS) prediction **(E)** by the E-signature. Significant survival difference between high- and low-threshold group. **(F)** A comparison with previously reported E-signature models by KM survival analysis and ROC curve. **(G)** The E-signature had the highest concordance index (C-index) as opposed to other reported models, which proved that it could accurately predict prognosis.

Based on multivariate Cox regression analysis, the independent prognostic signature still composed of four EMT genes was generated. Scoring formula as follows: Risk Score = 0.308×exp^DKK1^+0.299×exp^LOXL2^-0.084×exp^MGP^-0.165×exp^SLIT3^ (**Figure 4A**). The statistical correlation between risk model and 4 genes expression was assessed by the Pearson correlation metric (**Figure 4B**). Risk score was converted into Z-score, taking 0 served as the optimal boundary value to divide the samples into two groups, in which z score of the high-risk subgroup was greater than 0, and the rest belonged to the low-risk subgroup. The permutation of risk scores, survival status and four genes expression levels were displayed (**Figure 4C**). These results demonstrated that the risk E-signature was a deleterious indicator of prognosis.

Furthermore, based on TCGA training dataset, the prognostic accuracy of E-signature was appraised by time-dependent ROC analysis, the AUC was 0.73 (1-year), 0.7 (2-year) and 0.69 (3-year), respectively (**Figure 4D**). Referring to the training set, in comparison with the high-risk group, the low-risk group revealed a significantly longer OS (**Figure 4D**). The ROC and KM curve also revealed that our model exhibited good sensitivity and specificity in predicting TCGA-LUAD recurrence free survival (RFS, **Figure 4E**). Compared with recently published lung cancer prognostic models in the literatures (**Figure 4F**) including Zhu (51), Ma (52), Zhang (53) and Zhang M (54), our E-signature had better specificity by ROC curve analysis especially at 1-year (**Figure 4F**). Similarly, the E-signature predicted OS better than either the known signatures alone, with a better calibration and classification accuracy (**Figure 4G**).

### External validation of the prognostic E-signature

For further emphasizing the robustness of the constructed signature, GSE30219, GSE37745 and GSE50081 external validation sets were used for ROC analysis and KM analysis. In line with the training set, E-signature had strong predictive accuracy (GSE30219 [1-year: 0.74; 2-year: 0.69; 3-year: 0.73]; GSE37745 [1-year: 0.65; 2-year: 0.66; 3-year: 0.6]; GSE50081 [1-year: 0.72; 2-year: 0.68; 3-year: 0.68]) and patients in the group with high-risk showed a predominant association with frustrating OS (**Figure S2A**). Next, we opt GSE30219, GSE50081 and GSE8894 datasets and executed the identical methods to validate the prognostic potential of E-signature in RFS. Likewise, the E-signature maintained ideal sensitivity and specificity as a prognostic indicator (GSE30219 [1-year: 0.87; 2-year: 0.76; 3-year: 0.80]; GSE50081 [1-year: 0.67; 2-year: 0.68; 3-year: 0.68]; GSE8894 [1-year: 0.71; 2-year: 0.68; 3-year: 0.68]) and high-risk patients had significantly worse RFS relative to the group at low risk (**Figure S2B**).

### Establishment of a nomogram based on the E-signature

**Figure 5:**
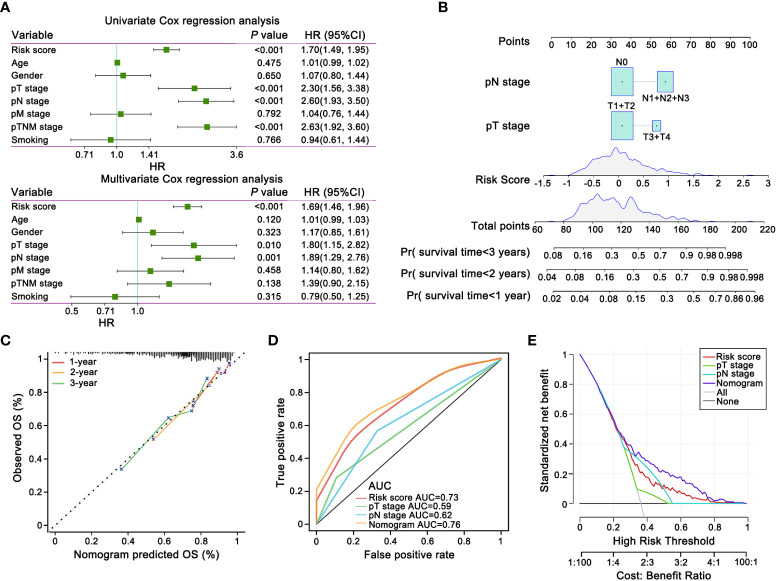
The prognostic nomogram and decision curve analysis (DCA) of the risk score based on E-signature. **(A)** The forest plot showed that the signature was independent from other risk factors for prognostic prediction. **(B)** Nomogram predicted risk of secondary progression. Each variable axis, containing T stage, N stage and risk score, corresponded to the characteristic attribute score of single sample. The final score was summarized on the total score axis and the likelihood of 1-, 2-, and 3 years OS is determined on the survival axis. **(C)** The nomogram yielded an accurate predictive capability that was extremely close to actual survival was presented by the calibration plots. X-axis represented the predicted value of survival probability and y-axis represented actual survival possibility. **(D)** ROC curves represented that the E-signature ranked first among all the parameters. **(E)** DCA curves graphically verified the E-signature brought more net benefit of survival than other clinical indexes. Solid lines indicated net benefit of the predictive model within the threshold probabilities range. The black and grey line respectively represented the hypothesis that none or all patients would experience.

clinicopathological characteristics, such as T stage, N stage, M stage, TNM stage, age, gender, and smoking history, and risk score were incorporated as covariates into univariate and multivariate Cox regression analyses. Risk score, T stage and N stage were demonstrated the independent prognostic factors by forest plot for OS (**Figures 5A**).

The nomogram could intuitively and effectively display the influence of the risk model on the prognostic outcome (55). A multi-scale nomogram was constructed on account of independent prognostic factors, which effectively predicted 1-year, 2-year and 3-year OS probabilities of LUAD patients. The scores corresponding to the parameters were calculated to obtain the total points. Thus, high total score was significantly correlated to worse outcome (**Figure 5B**). Furthermore, calibration curve implied well performance of the nomogram at predicting survival capacity in LUAD patients (**Figure 5C**). ROC curve analysis and Decision Curve Analysis (DCA) showed that, compared with other independent variables, the nomogram model classifier had the distinctly superior accuracy and net benefit rate (**Figures 5D, E**).

### Correlation between the prognostic model and clinicopathologic features

**Figure 6:**
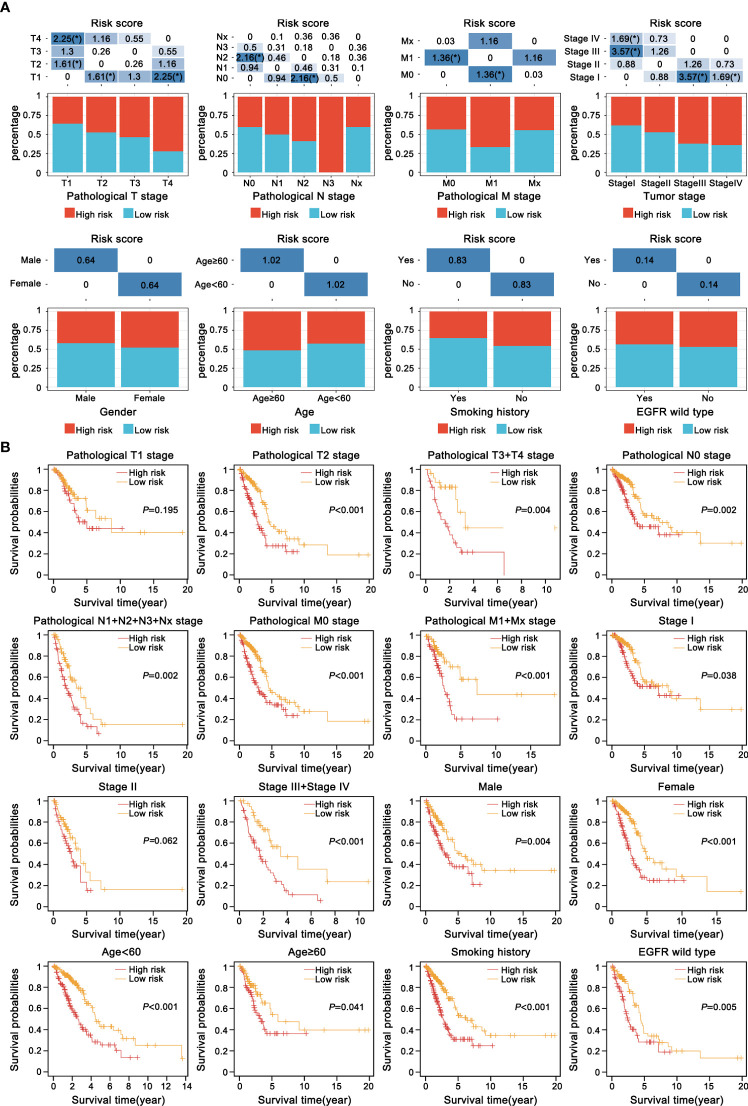
Correlation between the current prognostic E-signature and clinicopathologic attributes. **(A)** The distribution of samples with different risk scores classified by clinicopathologic traits, including T stage, N stage, M stage, tumour stage, gender, age, smoking history and EGFR mutation. *P < 0.05. **(B)** KM analysis of each subgroup was stratified by the E-signature to visualize the prognostic value of clinical parameters classification.

To further clarify the clinical implication of the EMT signature, we tested the relationship between risk score and clinicopathologic variables by the Chi-square test. LUAD patients were classified into different subgroups under diverse clinical properties, including T stage, N stage, M stage, tumor stage, gender, age, smoking history, status of EGFR. Notably, there was a gradual upward trend in the proportion of high-risk score patients with increase of malignant grade of pathological T stage, pathological N stage, pathological M stage and tumor stage. Regrettably, other clinicopathological characteristics showed no obvious difference in the distribution (**Figure 6A**). Subsequently, the risk model was also applied to each subgroup for KM survival analysis. We observed that subgroup with high-risk presented dramatically worse OS, suggesting that our specific signature had a precise predictive value (**Figure 6B**).

### Functional annotation and enrichment analysis of the E-signature

**Figure 7:**
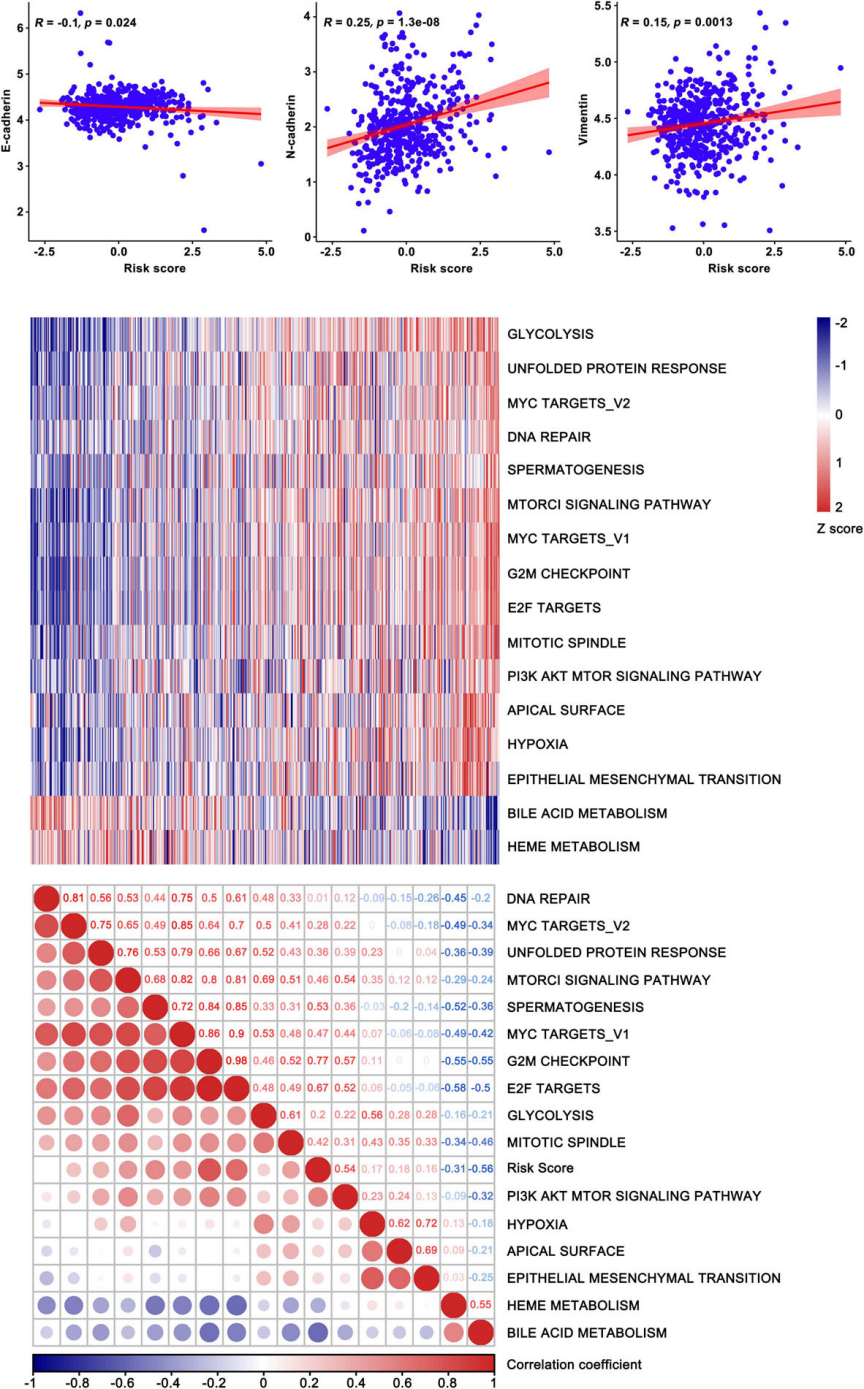
Functional enrichment analysis based on the current prognostic model. **(A)** The correlation between risk score and EMT biomarkers. **(B)** Hierarchical clustering analysis was used for heatmap plotting, showing KEGG pathways correlated with the model and coefficients were greater than 0.3. **(C)** The correlation map confirmed the high-risk group retained the oncogenic pathways. The risk score increased successively from left to right, where the enriched pathways and risk score were used as the abscissa.

The association between risk score and EMT biomarkers was assessed based on the TCGA database (**Figure 7A**). In order to further observe biological functions of the risk model, ssGSEA method was applied to derive scores of multifarious molecular pathways. The heatmap of hierarchical cluster analysis showed E-signature was enriched in “EPITHELIAL MESENCHYMAL TRANSITION” and other carcinogenic pathways, while metabolic-related pathways such as” HEME METABOLISM” and “BILE ACID METABOLISM” were inversely regulated by our signature (**Figure 7B**). Moreover, the correlation map visualized the KEGG pathways extracted by correlation coefficient > 0.3 statistically significant associated with the E-signature (**Figure 7C**). Taken together, the constructed specific signature played a compelling role in promoting tumor development.

## Discussion

LUAD is the accepted common classification of lung cancer, accompanied by high prevalence and fatality (2, 56). In cancer patients, metastasis is primary cause of shorten survival and high mortality, and often has already occurred at the time of diagnosis (57, 58). During the procedure of the classical invasion-metastasis cascade, transformation from tumor epithelial cells to mesenchymal cells with the invasion and migration capacity (59). Subsequently, mesenchymal cells locally invade the surrounding matrix and extracellular matrix (ECM), transport and stay in distant organ tissues, eventually extravasate and proliferate to form metastasis (60). The induction and ultimate success of this process depends on EMT and its key regulators (59). Regulation of EMT markers expression, for instance, N-cadherin, Vimentin and E-cadherin, ultimately affects tumor progression, metastasis and drug resistance (61, 62). To date, considerable studies have shown that the marked association of sophisticated regulation of EMT with poor prognosis of lung cancer patients (63, 64). To break down this barrier in clinical settings, molecular biological characteristics should be adequately considered, and circulating tumor markers, TNM staging and other indicators are not accurate enough in predicting the survival of LUAD patients (65, 66). Similarly, single-gene biomarkers are unable to achieve a satisfying prediction result (67). Therefore, a multigene panel might be a promising and reliable method for precision and individualized treatment of LUAD patients.

With rapid advancements in high-throughput sequencing, abundant studies have used data from large communal databases, for example, TCGA and GEO databases, to construct the prognostic signatures for identification of patient risk stratification, guidance of treatment regimens, precise prognostic assessment, and improvement of clinical efficacy (68, 69). Previous studies have reported risk-prediction models based on EMT in LUAD (37, 38). Although the conventional analytical methods used were similar, the genes required for construction of the signatures were fundamentally different, mainly due to the different databases and screening processes. In terms of bioinformatics analysis, our study not only plotted KM curves, ROC curves and nomogram to improve the accuracy and specificity of prognosis prediction, but also generated calibration curves for the nomogram to further prove effective clinical utility of E-signature. Secondly, the DCA (decision curve) was drawn for clarifying clinical feasibility of the risk model and prognostic significance of our signature was emphasized by comparing with known prognostic models (51–53). In addition, compared with the previously reported EMT models, we conducted *in vitro* experiments in-depth to verify a dramatically correlation between our signature and EMT procedure. Collectively, the specific E-signature we developed could predict the cancer process more comprehensively and accurately, with more stable and robust predictive performance and superior clinical practice (37, 38).

In this study, the E-signature was found to be composed of DKK1, LOXL2, MGP and SLIT3. It is reported that DKK1 (a secreted protein) contains the cysteine-rich domain and is a member of the family of Dickkopf, (70). DKK1 has emerged as an indispensable regulatory factor in multiple cancers and commonly existed as an inhibitor of the Wnt pathway (71–73). Nevertheless, DKK1 acting as a tumor promoter, which played a critical role in the cancer progression (74–76). LOXL2 belonging to the LOX family, has the typical function of catalyzing the cross-link of elastin and collagen in the ECM and has attracted much attention in cancer biology (48). Plenty of studies have revealed that LOXL2 participated in tumor progression, metastasis, poor prognosis and chemoradiotherapy resistance in varied cancers, e.g., lung cancer, breast cancer, pancreatic cancer and colorectal cancer (77–80). MGP, an extracellular matrix protein, whose well-defined function is still unknown, and currently acts as a double-edged sword in cancers (49, 81–84). Upregulation of MGP promoted cancer proliferation, migration and invasion, that was linked with unfavorable prognosis (49, 81–83). Whereas, MGP reversed chemotherapy resistance and received favorable survival outcomes in estrogen receptor positive breast cancer (84). SLIT3, a secreted protein, is widely distributed in normal tissues and mainly participates in the Slit/Robo pathway (85). SLIT3 has rarely been reported in human cancers, which could inhibit the progression of thyroid cancer (86).

## Conclusion

In summary, we developed and validated a trustworthy and powerful signature, which could serve as an independent and promising biomarker to optimize prognosis and surveillance protocols for individual LUAD patients.

## Data availability statement

The datasets presented in this study can be found in online repositories. The names of the repository/repositories and accession number(s) can be found in the article/**Supplementary Material**.

## Ethics statement

The study conducted in accordance with the guidelines of the Declaration of Helsinki, and was reviewed and approved by the Ethics Committee of Harbin Medical University. The need for written informed consent for participation was not required for this study in accordance with the national legislation and institutional requirements.

## Author contributions

LC and YX designed and supervised the research. YMC completed the first draft of manuscript. XW and JN collected pathological information of LUAD patients. YWC provided technical support for bioinformatics and RG carried out experiments, besides, WL offered auxiliary services. LZ performed statistical analysis and conducted the figures. YMC revised the manuscript and figures. All authors contributed to the article and approved the submitted version.

## Conflict of interest

The authors declare that the research was conducted in the absence of any commercial or financial relationships that could be construed as a potential conflict of interest.

## Publisher’s note

All claims expressed in this article are solely those of the authors and do not necessarily represent those of their affiliated organizations, or those of the publisher, the editors and the reviewers. Any product that may be evaluated in this article, or claim that may be made by its manufacturer, is not guaranteed or endorsed by the publisher.
