# Supplementary material for: A novel epithelial-mesenchymal transition (EMT)-related gene signature of predictive value for the survival outcomes in lung adenocarcinoma
Source: Front Oncol. 2022 Sep 15;12:974614. doi: 10.3389/fonc.2022.974614 (PMC9521574; doi:10.3389/fonc.2022.974614)
Supplement: Supplementary file 1 [file DataSheet_1.docx]

Supplementary Material

## Supplementary Figures


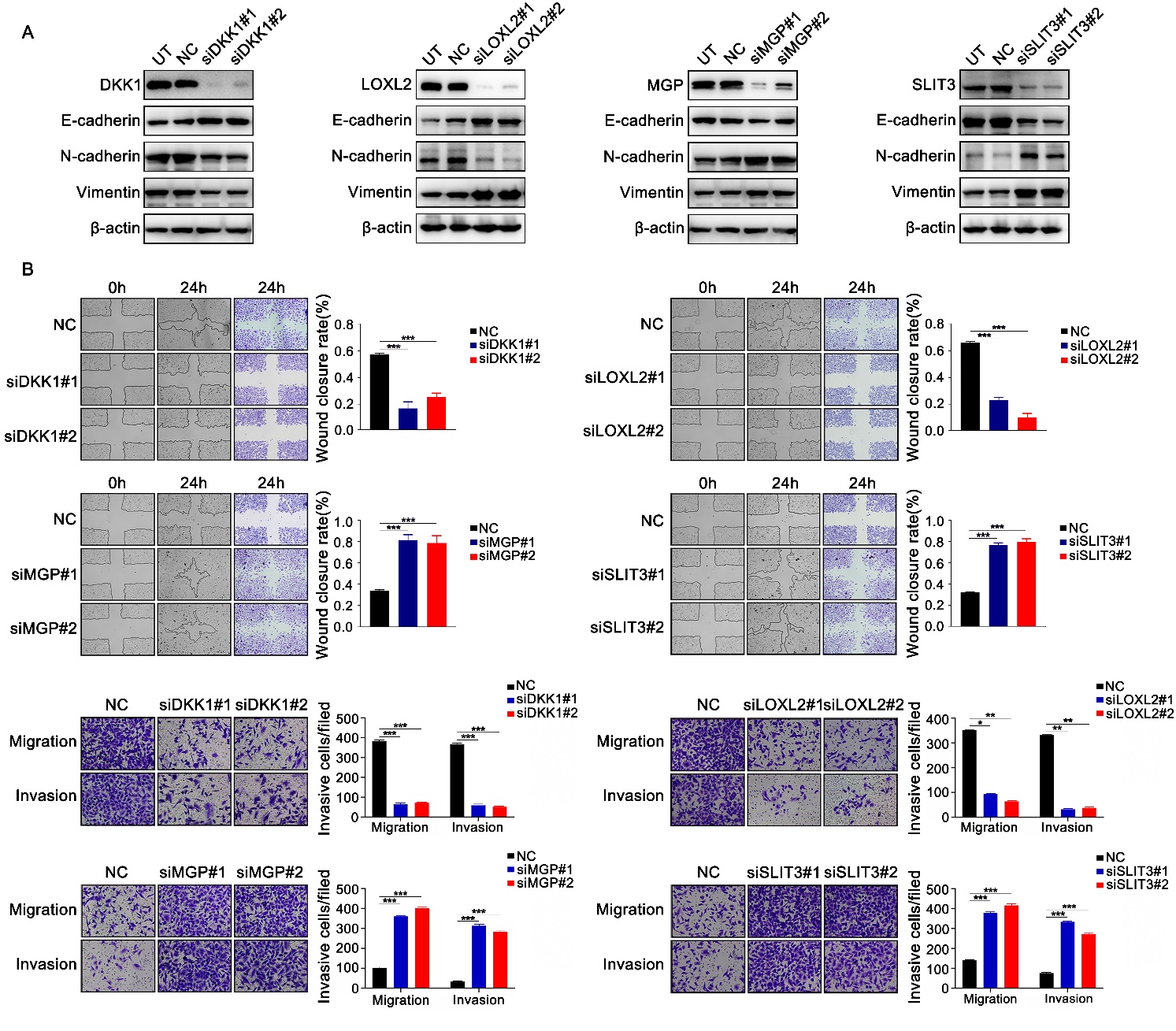
 **Supplementary Figure 1.** The validation with *in vitro* experiments of 4 prognostic EMT-related genes comprising the E-signature. **(A)** Western blot confirmed that silencing expression of 4 genes respectively caused changes of the EMT markers expression in A549 cells. **(B)** The influence of silencing the four genes expression exerted on migration of A549 cells. **(C)** Transwell assays investigated the migration and invasion of A549 cells (**P* < 0.05; ***P* < 0.01; ****P* < 0.001).


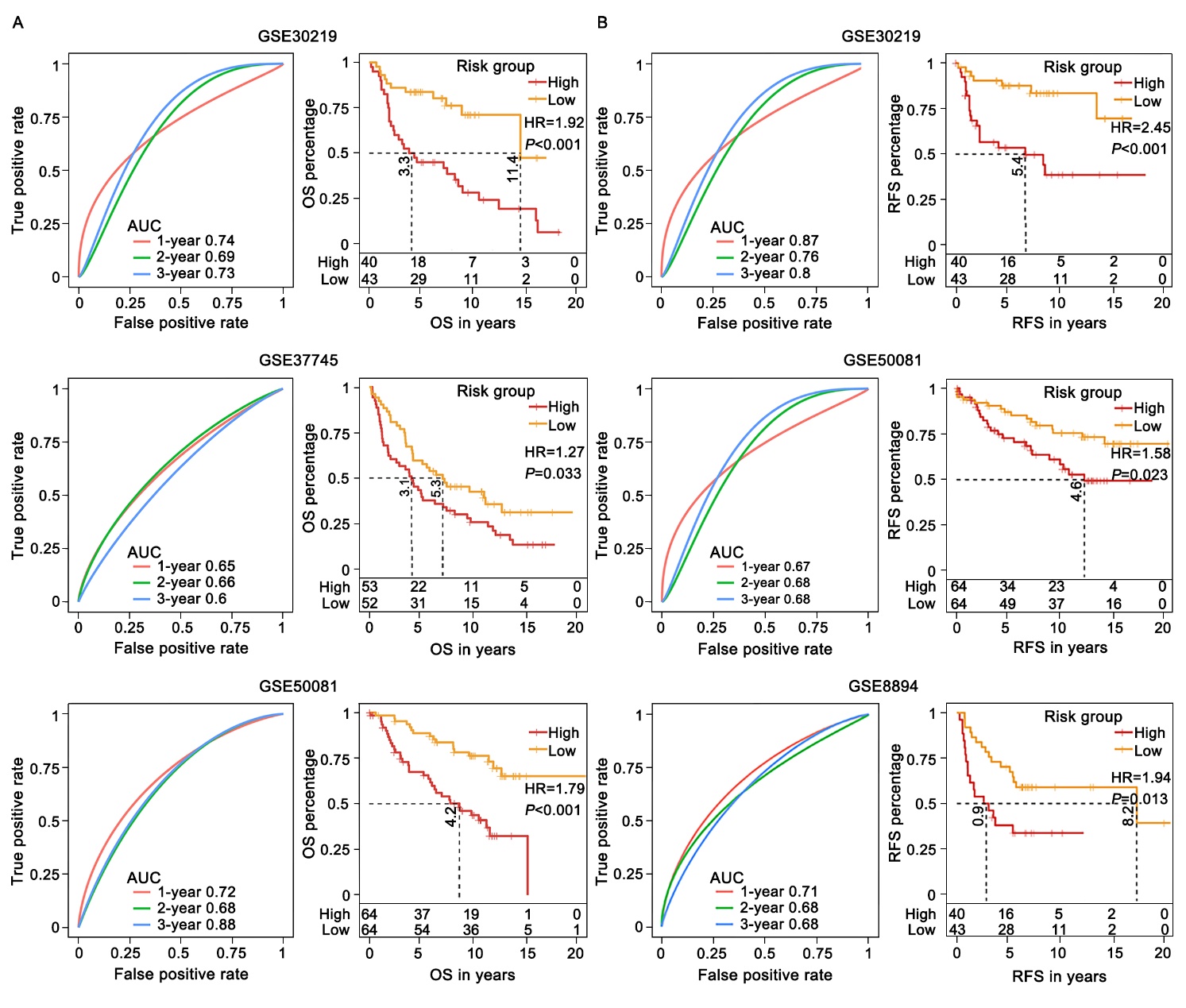


**Supplementary Figure 2.** The prognostic robustness and clinical usefulness of the E-signature in the validation sets. **(A)** External datasets verified predictive OS performance of the risk signature by ROC curves and performing KM survival analysis, including GSE30219, GSE37745 and GSE50081. **(B)** AUC of the ROC curves was computed under diverse datasets, including GSE30219, GSE50081 and GSE8894. RFS differed greatly based on the risk score gained from the E-signature.
